# Pediatric Trauma Care in Low Resource Settings: Challenges, Opportunities, and Solutions

**DOI:** 10.3389/fped.2018.00155

**Published:** 2018-06-04

**Authors:** Andrew W. Kiragu, Stephen J. Dunlop, Njoki Mwarumba, Sanusi Gidado, Adesope Adesina, Michael Mwachiro, Daniel A. Gbadero, Tina M. Slusher

**Affiliations:** ^1^Department of Pediatrics, Hennepin Healthcare, Minneapolis, MN, United States; ^2^Department of Emergency Medicine, Hennepin Healthcare, Minneapolis, MN, United States; ^3^Division of Global Medicine, University of Minnesota, Minneapolis, MN, United States; ^4^Department of Political Science, Oklahoma State University, Stillwater, OK, United States; ^5^Department of Surgery, Bingham University Teaching Hospital, Jos, Nigeria; ^6^Department of Surgery, Bowen University Teaching Hospital, Ogbomosho, Nigeria; ^7^Department of Surgery, Tenwek Hospital, Bomet, Kenya; ^8^Department of Pediatrics, Bowen University Teaching Hospital, Ogbomosho, Nigeria; ^9^Division of Global Pediatrics, University of Minnesota, Minneapolis, MN, United States

**Keywords:** low- and middle-income countries, trauma, pediatrics, injury prevention, emergency management, surgical management, child abuse, disasters

## Abstract

Trauma constitutes a significant cause of death and disability globally. The vast majority -about 95%, of the 5.8 million deaths each year, occur in low-and-middle-income countries (LMICs) 3–6. This includes almost 1 million children. The resource-adapted introduction of trauma care protocols, regionalized care and the growth specialized centers for trauma care within each LMIC are key to improved outcomes and the lowering of trauma-related morbidity and mortality globally. Resource limitations in LMICs make it necessary to develop injury prevention strategies and optimize the use of locally available resources when injury prevention measures fail. This will lead to the achievement of the best possible outcomes for critically ill and injured children. A commitment by the governments in LMICs working alone or in collaboration with international non-governmental organizations (NGOs) to provide adequate healthcare to their citizens is also crucial to improved survival after major trauma. The increase in global conflicts also has significantly deleterious effects on children, and governments and international organizations like the United Nations have a significant role to play in reducing these. This review details the evaluation and management of traumatic injuries in pediatric patients and gives some recommendations for improvements to trauma care in LMICs.

## Introduction

The global emphasis on reductions in childhood mortality and meeting the Sustainable Developmental Goals (SDGs), has resulted in significant gains in reducing childhood deaths around the world (1). However, an epidemiologic shift has been noted, with relative increases in deaths from injuries and declines in deaths from poor nutrition and infections such as pneumonia and diarrheal diseases (2).

**Figure 1:**
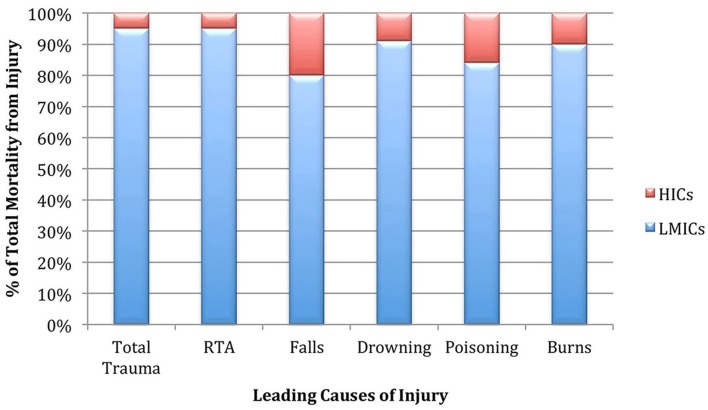
Illustration of the burden of trauma-related mortality borne by LMICs in comparison to HICs.

Trauma constitutes a significant cause of death and disability globally. About 95%, of the 5.8 million deaths each year occur in low-and-middle-income countries (LMICs) (3–6). Almost 1 million of these deaths are children. The World Health Organization (WHO) reports that the top five etiologies for unintentional injuries are road traffic accidents (RTAs), falls, burns, drowning and poisoning (3–6) (Figure 1). An alarming number of children are also injured or killed in war-zones, in disasters, and from child abuse (3, 7). Resource limitations in LMICs necessitate trauma prevention and thoughtful resource allocation and utilization in the care of injured children. This review details the evaluation and management of pediatric trauma in LMICs.

## Emergency response to trauma

### Pre-hospital systems and triage

In high-income countries (HICs), caring for injured patients involves well-coordinated systems of triage, emergency medical care, and critical care. While such systems are currently unfeasible in many LMICs, it is essential that capabilities for managing acute onset, severe but reversible disease and injuries are available in any country around the world (8).

Wholesale, poorly planned imitations of HIC-type pre-hospital systems in LMICs often result in expensive, ineffective systems (9). There are, alternatively, low-cost interventions, for example, first responder training programs in Uganda and Mexico. These have resulted in excellent outcomes for relatively low costs (9). In LMICs, successful systems of pre-hospital trauma care take into careful consideration local financial resources and capitalize on them and also are broadly acceptable in the local societal context (9). Initiatives to improve or implement pre-hospital or trauma systems in LMICs must recognize domestic resource constraints to minimize financial strain and improve efficiencies in the distribution of these resources (10). Emergency care depends on recognition of severe injury or illness and timely intervention. It involves the ability to quickly obtain care, rapid and appropriate referrals and the safe transportation of patients (11). The absence of a formal triage system in many hospitals in LMICs often leads to potentially life-threatening delays in obtaining needed care for patients who are severely injured or critically ill (12). The WHO, to bridge this gap in pre-hospital care that is present in many LMICs, recommends the establishment of first-responder programs to train laypeople as the first step toward building pre-hospital systems in LMICs (13). There are examples of such programs that have successfully used local resources to educate laypersons with little formal education (14). Effective triage and emergency care have also been described in some LMICs. The South African Triage Scale (SATS) for children is one such tool. It is used to prioritize children requiring emergency treatment. By employing a triage early warning score in combination with clinical signs and symptoms, the SATS helps to identify acute illness earlier, improves emergency department patient flow and allows better stewardship of hospital resources (15).

### Emergency department management

**Figure 2:**
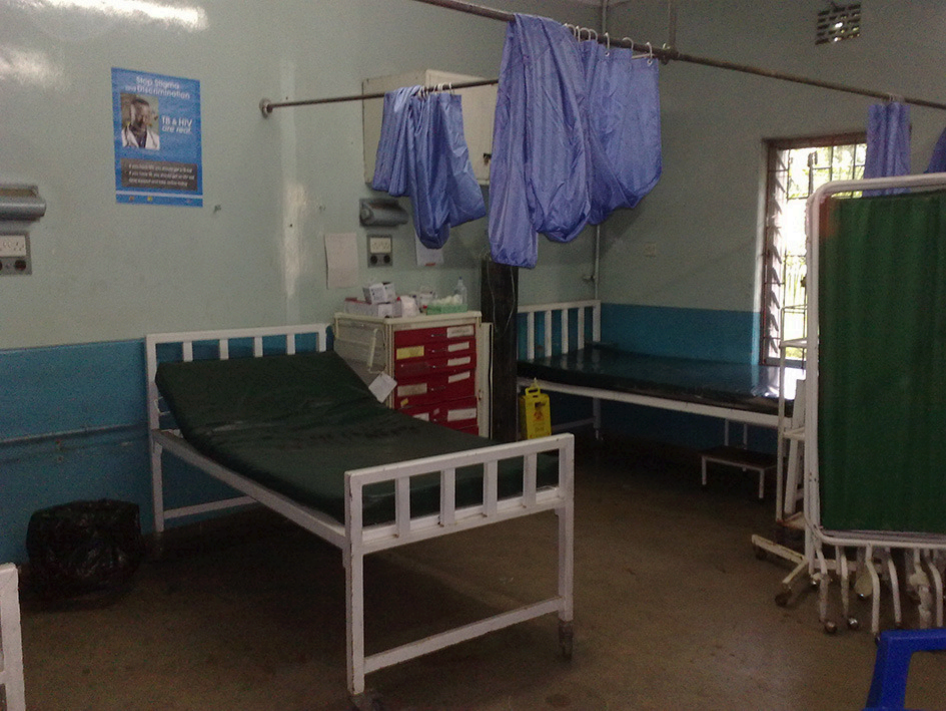
Example of an Emergency Room in a public hospital in a LMIC. Courtesy Benjamin Wachira, MD Aga Khan University Hospital, Nairobi Kenya.

Inadequate staffing levels coupled with huge patient loads lead to delays in assessment and treatment in many hospitals in LMIC's (16). While most hospitals have a dedicated emergency or casualty department, few have emergency medicine-trained specialists. Even more rare are dedicated trauma centers. This means that healthcare providers with no specialized training in the management of pediatric trauma provide the majority of pediatric trauma care. This has a significantly negative impact on outcomes, which are dependent more on the speed and appropriateness of the medical care received than how severe of an injury was sustained (17). There are significant differences even within the same LMICs with regards to available resources for emergency room care between public and private hospitals (Figure 2). Public hospitals, which usually have fewer resources, are often overwhelmed. A recent study of 7 EDs in Pakistan noted that on average, only about 17% of patients were appropriately triaged, and fewer than 25% had any vital signs documented (18). To improve patient flow in overcrowded EDs, a recent study employed LEAN methodologies in a teaching hospital in Ghana (19).

Like the previously noted SATS, the Emergency Triage, Assessment, and Treatment plus (ETAT+) training in LMICs has resulted in better prioritization of pediatric trauma cases in the ED (20). Improved outcomes have also been achieved in some institutions in LMICs that have developed protocols for trauma management (21–25). Comprehensive Advanced Life Support (CALS®) and Advanced Trauma Life Support (ATLS)® are two examples of guidelines for the management of trauma. Organizations such as the African Federation of Emergency Medicine (AFEM) and the WHO have also provided guidelines on the appropriate resources needed for the care of pediatric trauma patients which are adjustable based on local resources (13, 22–25). Given the expense associated with maintenance of CALS and ATLS training, locally developed standardized trauma protocols have been found to be effective in achieving increased use of timely appropriate interventions for trauma patients and associated with decreased mortality rates particularly in patients with severe traumatic brain injuries (26).

### Surgical management

Surgical management is the cornerstone of trauma care. In HICs the ready availability of multispecialty surgical teams as key members of the trauma management team facilitates timely surgical intervention when needed and the improved outcomes that this translates to. Given the lack of even basic surgical services in many LMICs, the surgeon-led trauma team and related resources remain a dream in most low-resource areas of the world. Globally, an estimated 2 billion people lack access to even the most basic surgical care (5). A recent study by Higashi et al. found that 1 million deaths and the loss of 52.3 million DALYs could have been averted in all LMICs if a basic menu of surgical services were made universally available (27). These services included basic resuscitation, advanced life support including the provision of surgical airways, peripheral venous access, laceration, and wound management, needle decompression and chest tube placement, fracture reduction, escharotomy and fasciotomy, skin grafting and trauma-related laparotomies (27). Governments in LMICs in conjunction with international partners have a significant role to play. Many essential physical resources, including equipment and supplies, are low cost and can be better supplied through improved planning and logistics (5). Enhanced durability, lower purchasing and operating costs as well as increased capabilities for local manufacture, maintenance and repair could enhance the availability of more expensive equipment like x-ray machines and ventilators (5). The poorest LMICs will require international assistance for the initial purchase of basic essential equipment and supplies (5). In the interim, surgical services in some LMICs, particularly during crisis situations, have been provided by non-governmental organizations (NGOs) (5). This assistance has varied from short-term mission trips by groups like Operation Smile to mobile, self-contained surgical platforms provided by Médecins Sans Frontières that remain in-country for months to years (5). There are also examples of more permanent specialty surgical hospitals established by NGOs in-country (5).

Trauma teams adapted to local conditions and resources in each LMIC should be developed. This will also require financial commitments to facilitate training and equipment purchases (28). Tele-simulation is another option for teaching and developing pediatric trauma resuscitation skills to healthcare providers in LMICs (25). An excellent resource for online learning and simulation is OPEN Pediatrics, an online community of clinicians that share best practices from all around the world (29). Incorporation of trauma management training into the undergraduate medical school curriculum will help ensure on-going widespread dissemination of the skills required to manage pediatric trauma.

## Addressing specific causes of pediatric injury

### Road traffic injuries

Each year there are over 1 million deaths associated with RTAs and an estimated 20–50 million non-fatal road traffic injuries (RTIs) around the world (30). These RTAs result in the deaths of 186,300 children (ages 0–17 years) (31). Boys are twice as likely to be killed in RTAs as girls. LMICs account for approximately 95% of all children killed by RTAs, with the brunt of this burden borne by countries in Sub-Saharan Africa. Over 35% of global child deaths from RTAs are in Sub-Saharan Africa (31). To address this significant problem, several steps should be taken. These include building road safety management capacity, improved infrastructure, and enhanced vehicle safety. Also, improved road user behaviors with enforcement of speed limits, seat belt and laws prohibiting driving under the influence of alcohol could also make an impact (30, 31). Improved pre-hospital, hospital and rehabilitation systems would help reduce the mortality and morbidity associated with RTIs (31).

### Falls

Over 400,000 fatal falls occur each year globally. This makes them the second-leading cause of unintentional injury-related deaths after RTAs. More than 80% of fall-related fatalities occur LMICs (32). While not all fall injuries are fatal, every year more than 37 million fall-related injuries are severe enough to require medical attention. They account for over 17 million DALYs (32). Children living in countries with poor infrastructure and unsafe housing conditions are especially at risk for injuries from falls (3). Other fall risk factors include male sex and age. In LMICs infants have significantly higher rates of fall-related injuries than older children (5). Efforts to prevent falls include developing and promoting local manufacture of inexpensive measures to prevent falls such as window guards, building regulations and enforcement that prevent unsafe housing, access to safe playgrounds, and better supervision of children (5).

### Drowning

According to the WHO, worldwide there are 370,000 deaths from drowning making it the 3rd leading cause of unintentional injury-related mortality. Ninety-one percent of these deaths occur in LMICs (33). Children at the greatest risk of drowning are unsupervised boys in rural areas with little formal swimming instruction. Strategies to prevent drowning include placing barriers around bodies of water, covering wells, increased supervision, providing formal swimming lessons, and increasing community awareness about the risks of drowning (33, 34).

Comprehensive boating regulations and enforcement, improved signage including designation of dangerous water bodies, enhanced water rescue and resuscitation, water safety requirements including the use of personal flotation devices and improved supervision of swimming areas used for recreation are also important (34).

### Poisoning

Poisoning is a leading cause of morbidity and mortality globally. There are multiple causes of poisoning ranging from pesticides and industrial chemicals to lead and mercury poisoning. LMICs bear the larger part of the burden with regards to poisoning. According to WHO data, in 2012, over 190,000 people died worldwide from unintentional poisoning. Of these deaths, 84% occurred in LMICs (35). Unintentional poisoning also resulted in the loss of over 10.7 million years of DALYs (35).

Poverty, lack of education, poor quality controls and absent legislation regarding certain products are some of the challenges that exacerbate this problem. Suicide from intentional poisoning point to the significant challenge of mental illness in LMICs and the inadequate resources available to combat this problem (35, 36). Nearly a million people die each year as a result of suicide, and chemicals account for a significant number of these deaths. Deliberate ingestion of pesticides causes approximately 370,000 deaths each year, and self-poisoning is the most common method of suicide attempt in youth in LMICs (35, 36).

## Evaluation and management of specific system-based injuries

### Traumatic brain injuries

Traumatic brain injuries (TBIs) constitute a significant public health problem and a leading cause of death and disability worldwide (37, 38). They affect over 3 million children annually, impacting every population and demographic group (38). RTAs are the leading cause of TBIs worldwide followed by falls (38). The vast majority of TBIs occur in LMICs where inadequate pre-hospital and hospital-based care and poor rehabilitation facilities result in unsatisfactory outcomes (37, 39). The mortality rate for TBI patients in LMICs is twice that in HICs (40) with most of these deaths being of patients with severe TBI (40).

There are recently published guidelines for the management of severe traumatic brain injury in infants and children (41). However, the dearth of resources in LMICs means that the application of these guidelines is variable (37). Given the limited neurosurgical and neurocritical care capacity in most LMICs, consideration should be given to regionalization to optimize neurosurgical resources. Resource-adapted courses similar to the Advanced Life Support in Brain Injury (ALSBI) course may lead to broader dissemination of the knowledge needed to improve outcomes from pre-hospital to rehabilitation (42).

Most TBIs in children are mild with Glasgow Coma Scale scores of ≥13. These children usually have no significant findings on radiographic evaluation (38). In situations where the injuries are more severe, the most common findings are skull fractures, brain parenchymal hemorrhages, and contusions (38). Unfortunately, these patients have poorer outcomes particularly in LMICs where neurosurgical intervention is often unavailable (38).

### Management of TBI

The goal of TBI management is to treat the primary injury, when indicated, including the evacuation of subdural and extradural hematomas and repairing significant skull fractures. Treatment is aimed at preventing any secondary insults to the brain through improvements in cerebral perfusion and provision of adequate oxygen (41, 43). To achieve this, placement of an advanced airway and mechanical ventilation may be required. The capacity to do this is absent in many LMIC settings where even provision of supplemental oxygen might be difficult. To maintain cerebral perfusion, patients often need intravenous fluids and sometimes might require initiation of vasopressor agents. In patients with a severe TBI defined as a GCS ≤ 8, the optimal evaluation would ideally include a head computed tomography (CT) to visualize any skull fractures and intracranial pathology (44, 45). In many LMICs, CT scanners are often not available or are out of the financial reach of most of the general population. In such situations, there may be some utility to skull x-rays which can at least identify fractures but do not reveal intracranial injuries (45, 46). In situations where clinical examination or, when available, intracranial pressure monitoring, reveals increased intracranial pressure and evidence for cerebral edema, hyperosmolar therapy is initiated (41, 43). The hyperosmolar therapy used in LMICs is most often mannitol although in some countries hypertonic saline is also used (41, 43). Ventriculostomy catheters to monitor ICP are usually indicated when the GCS ≤ 8. In many resource-limited areas, the capability to do this is frequently absent. Ultrasonographic optic nerve sheath diameter measurement can be used to detect elevated ICP, although in most low-resource settings the equipment and expertise to perform this evaluation are lacking (47). Nonetheless, ultrasound is available in many hospitals thus making this training possible and potentially very valuable. The BESTTRIP study offers an example of how to manage patients when a ventriculostomy is not available (48). This study revealed no substantial difference in outcome between patients with invasive monitoring of ICP and those evaluated with clinical exams and repeat CT scans (48). Unfortunately, this would be difficult to replicate in most LMICs. In HICs the ready availability of neurosurgical expertise means that any required surgical interventions such as evacuation of subdural and extradural hematomas and decompressive craniectomy for ICP elevation unresponsive to medical therapy are easily accomplished. In LMICs this is significantly more difficult to achieve. Other aspects of care that could be accomplished even in low resource settings include elevation of head-of-bed, maintenance of euthermia, provision of adequate pain control and sedation, provision of adequate nutrition and seizure prophylaxis and control (40, 41, 43).

### Abdominal injuries

Abdominal injuries (AI) are associated with a significantly increased risk of death and disability especially when other injuries and in particular TBI are present (49). The majority of AI result from motor vehicle-related crashes and falls (49, 50). Clinical signs and symptoms concerning for AI include abdominal tenderness and distention, absent bowel sounds and peritoneal signs. The presence of the latter may indicate a need for surgical exploration (49, 51). In children who present with the classic abdominal wall bruising consistent with a seat-belt injury, a high index of suspicion for bowel, kidney, and vertebral injuries is required (50). Splenic and hepatic injuries are the most frequently noted AI's. A system of classification established by the American Association for the Surgery of Trauma (AAST) for these visceral injuries helps guide prognosis and management (52). Bowel injuries are found in approximately 1–5% of blunt abdominal injuries (49). Most commonly injured is the jejunum, which accounts for about 30% of all hollow-viscous injuries. The second most commonly injured part of the bowel is the duodenum (49).

### Management of abdominal injuries

Initial management of an AI involves ensuring a patent airway, adequate oxygenation, and IV fluid resuscitation if indicated (49). Ongoing evaluations including of vital signs, neurologic and abdominal exams and urine output are important (49). In HICs, initial imaging after an AI involves a Focused Assessment with Sonography for Trauma (FAST) evaluation (49). This helps to identify intraperitoneal blood or fluid from a visceral injury (49). However, FAST exams potentially miss about a third of visceral injuries in children (50). A potential impediment in LMICs is that ultrasound machines are not always available and expertise in their use is variable. CT scans are standard in the evaluation of abdominal trauma in HIC's but less likely to be available in LMICs (51). Ultrasonography is, however, despite the limitations noted, more readily available and additional training in ultrasound use is invaluable to clinicians in these settings. In HIC's, over 90% of blunt AIs are managed non-operatively (49). However, non-operative management has been made possible by advanced imaging techniques that are limited in LMIC's which itself has led to higher rates of surgical exploration (50). Otherwise, clinicians must rely on their physical diagnostic skills in combination with peritoneal taps. If urgent surgical intervention is needed in these patients, then transfer to centers offering a higher level of surgical and critical care should be done expeditiously as possible once resuscitation has begun.

### Thoracic injuries

Thoracic injuries (TIs) constitute an ongoing challenge to the trauma or general surgeon practicing in LMICs and have associated high morbidity and mortality (53). The majority of these are blunt thoracic injuries and most often results from RTAs. Penetrating trauma is mainly related to gunshot wounds and other projectiles (54). Young children have more compliant, cartilaginous chest walls and therefore even significant force injuries are less likely to result in fractures. However, there is a greater transmission of these forces to the child's internal organs and these patients will still have associated pulmonary and cardiac contusions, pneumothoraces, hemothoraces and mediastinal injuries (54, 55). Given the disproportionately smaller size of the thorax in comparison to the cranium and abdomen, it is imperative to assess the entire patient when thoracic trauma is present to rule out TBIs and abdominal injuries (56–59).

### Management of thoracic injuries

**Figure 3:**
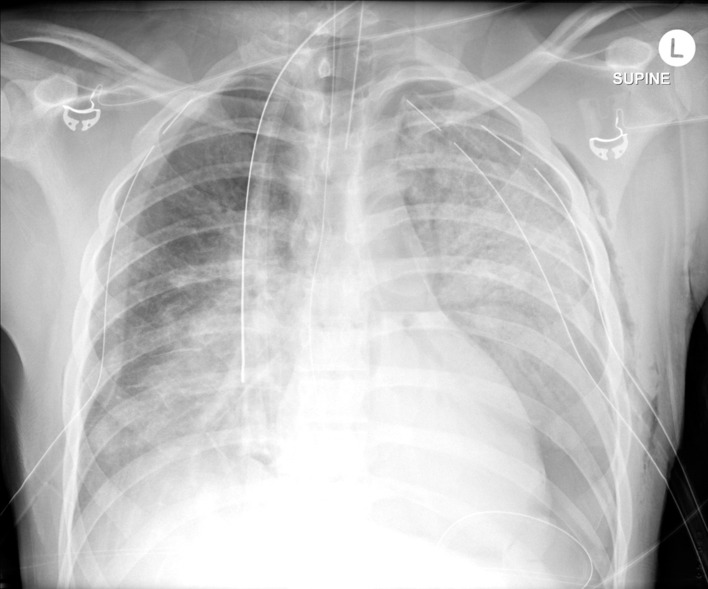
Chest x-ray showing bilateral pulmonary contusions and pneumothoraxes requiring chest tube placement.

Evaluation of thoracic injuries begins with primary and secondary surveys. In addition to the FAST exam, a basic x-ray of the chest is required. CT scan imaging and other advanced imaging techniques can be useful adjuncts but are frequently not readily available in many LMICs. Again, where available the value and use of ultrasound should be capitalized on, and the FAST exam taught. They are also a strain on resources for both patients and healthcare facilities and increase radiation exposure to children (57, 60). Initial resuscitation should follow the usual trauma protocol attention to airway, breathing, and circulation (ABCs) while ensuring C-spine immobilization for polytrauma patients. Clinical presentation of thoracic injuries is dependent on the type of injury. An underlying pulmonary contusion is usually more prognostic than the chest wall injury itself (56). (Figure 3) Rib fractures are usually very painful because of the inability to immobilize them. The extent of the multisystem injury is directly proportional to the number of rib fractures. Scapular, clavicular and rib 1-3 fractures are linked to cardiovascular injury and indicate a high-energy mechanism (61). A high index of suspicion for traumatic asphyxia for patients who present with tachypnea and facial petechiae is important (56). Children are more likely to develop hypoxia than adults due to their lower functional residual capacity and relatively higher tissue oxygen consumption (54). Additionally, their more mobile mediastinum allows for the faster conversion of a simple pneumothorax to a tension pneumothorax (54). Proper airway management takes priority in patients with tracheobronchial injuries. Other more rare injuries include esophageal injuries, traumatic diaphragmatic rupture, and cardiac injuries. A plain chest x-ray can diagnose diaphragmatic injury with herniated viscera, and esophageal tears commonly with left sided pleural effusions (54). Algorithms have been developed for these that can be adapted to the LMIC setting (55). Blunt cardiac injury (BCI) is often under-diagnosed due to a lack of diagnostic tools including troponin laboratory screening, electrocardiography, and echocardiography, as well as a low index of suspicion (62, 63). Persistent tachycardia or other arrhythmia in the face of thoracic trauma should prompt an evaluation for BCI. In HIC, emergency thoracotomy has been described and has been shown to be lifesaving for children with penetrating cardiac injuries (64, 65). Indications for ER thoracotomy are well defined and include massive hemothorax, initial chest tube output >20 mls/kg, and pericardial effusion on ultrasound in the setting of shock (66). In many LMIC settings, the absence of equipment and trained staff means that ER thoracotomy is not currently feasible. However, temporizing measures such as pericardiocentesis or pericardial catheter placement may be possible. Whenever possible, early referral to a better-equipped trauma unit should be made after initial stabilization.

### Orthopedic injuries

**Figure 4:**
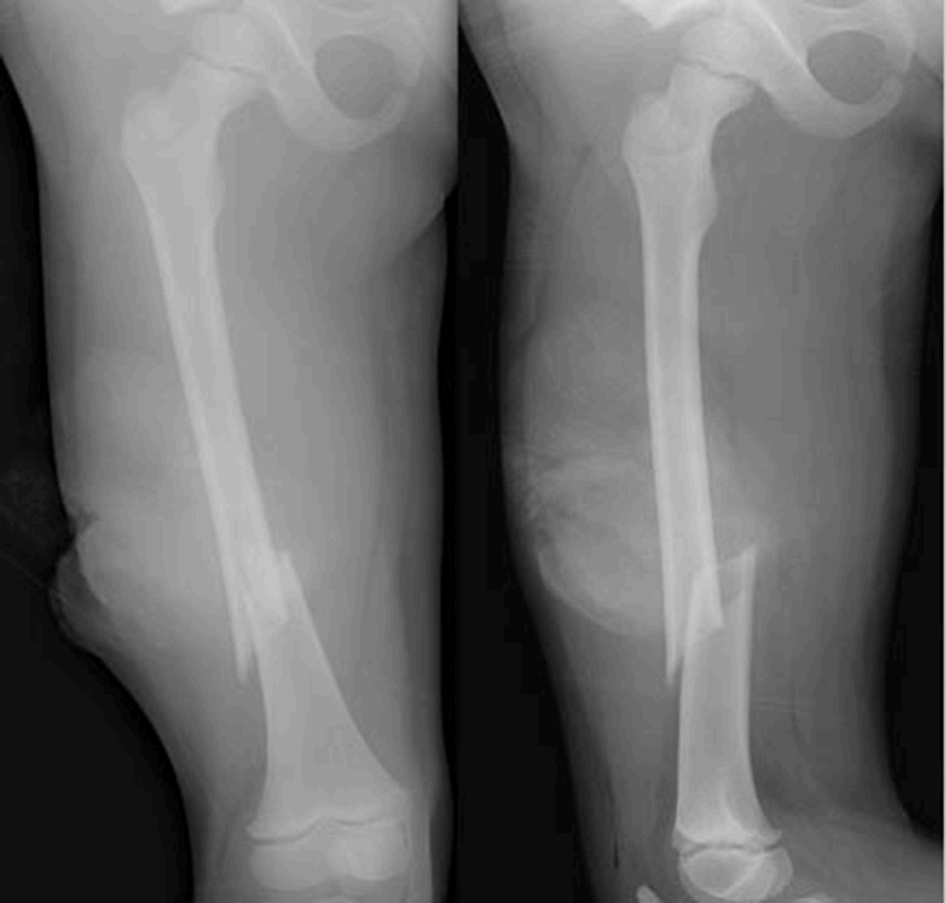
X-ray showing right femur fracture.

Orthopedic injuries have a significant impact on DALYs in LMICs since those most commonly injured are typically younger, potentially more productive persons (2, 3, 67). The causes of these injuries include falls, RTAs, workplace accidents, child abuse, and injuries sustained in conflicts or other disasters (1–3). Fractures in pediatric patients are distinct from those in adult patients. Young children have a growth plate, and physeal fractures represent ~18% of fractures (68). Four distinct types of fractures seen in children include plastic deformity, torus fractures, greenstick fractures, and physeal fractures (68). Thoughtful evaluation with attention to the child's neurovascular status is key. Radiologic evaluation of the fracture when radiographic equipment is available requires at least two planes, including the joint above and below, if necessary (68) (Figure 4).

### Management of orthopedic injuries in LMICs

Management of these fractures begins with stabilization of the fractured extremity. Consideration may be given to pelvic binding, if indicated for hemodynamic stability, and should be performed as part of the circulation assessment in the primary survey. Any open wounds should be irrigated with large amounts of sterilized water, and antibiotic therapy started if indicated, in addition to tetanus prophylaxis (69, 70). Adequate analgesia is important in children. This can vary from acetaminophen and other non-steroidal anti-inflammatory drugs for mild pain to opioids for more severe pain.

Management is conservative in closed uncomplicated fractures. External fixation and wound care for open fractures, and open reduction and internal fixation in selected cases may be required. In HICs, interdisciplinary management decisions are made by orthopedic surgeons in collaboration with plastic, vascular, trauma, and general surgeons as well as physiotherapists and occupational therapists (71). However, in many LMICs, it is difficult for most patients to access this interdisciplinary care.

A large burden of care in these countries is borne by general surgeons as there is not a large pool of orthopedic surgeons and even fewer vascular or plastic surgeons. The surgeon is faced with patients whose initial care is not by trained first responders (72). Sometimes the surgeon may care for patients who elected to initially be managed by “traditional bone healers.” There is also delay in obtaining consent from families for any procedures other than initial stabilization and wound care (73). There is, therefore, a higher rate of limb amputations from orthopedic injuries. This increases costs and length of stay due to additional care needs and rehabilitation (73). The surgical care of orthopedic injuries is increasingly being recognized as a more cost-effective modality of treatment than more conservative methods in many LMICs. Previously, surgeons improvised with old-fashioned implants and equipment donated to their hospitals from HICs with varying results. More recently, there are centers in LMICs that have access to appropriate implants and training through programs like SIGN Fracture Care International. This has resulted in fracture treatments and outcomes that are comparable to those in HICs (74). One impediment to appropriate orthopedic care is the fact that most orthopedic surgeons are located in urban areas with practices that are out of the reach of most of the general population. Governmental policies and financial investments are needed to facilitate the training of more surgeons in all specialties, and making these surgeons available and accessible at most public access hospitals. General surgeons in LMICs should also receive more training in the management of orthopedic injuries. Consideration should also be given to providing additional training in basic orthopedic stabilization, to nurses and medical officers who typically are the initial healthcare providers for injured children in most public access facilities in LMICs.

### Burn injuries

Burns, especially those that leave a child permanently disfigured or disabled, represent the most catastrophic events to happen to a child (75). Globally, over 11 million people are estimated to suffer burn injuries leading to over 265,000 deaths annually (75, 76). As with other traumatic injuries, the vast majority of burn injuries and deaths occur in LMICs (76, 77). Children <5 years of age are usually at the greatest risk for burn injuries, with an estimated 100,000 admissions annually. This number is likely a significant underestimation (78). In HICs, improvements in burn management have led to decreases in morbidity and mortality with most burn centers in HICs reporting an LA50 (lethal total body burn surface area [TBSA] for 50% of patients) >90% TBSA (79). These improvements have not universally been seen in LMICs where there is large variability in outcomes, with most centers reporting an aggregate LA50 <40% and many reporting 100% mortality with burns >40% TBSA (80). If LMIC center outcomes matched those in the best performing HIC centers, over 34,000 additional lives could be saved worldwide (81). These poor outcomes are multifactorial but are most often related to delayed presentation, lack of trained personnel and a paucity of burn centers (80). One regional referral center in Tanzania found that almost half of burns arrived >72 h after injury (82).

### Management of burns in LMICs

Management of burn injuries includes the early recognition of major burns (>10–20% partial thickness and full thickness burns), evaluation and management of airway involvement, provision of oxygen and recognition of carbon monoxide and cyanide poisoning, and fluid resuscitation for management of burn shock.

A survey of burn resuscitation in the African continent revealed that parenteral fluid resuscitation protocols using lactated Ringers solution based upon the Parkland formula are the most commonly utilized (83). There was also an increased utilization of enteral hydration in the form of oral rehydration solution (ORS), along with a focus on clinical endpoints such as urine output, rather than invasive monitoring in comparison with higher resourced counterparts. ORS is a viable option for burn resuscitation for burns <20% TBSA (84). Patients with significant burns have better outcomes when treated at centers with expertise in burn care, and therefore, after stabilization immediate transfer whenever possible is encouraged. These centers, employ typical burn therapies such as protocolized resuscitation, topical antibiotics, skin grafting and have a mean daily cost per 1% total burn surface per patient as low as $2.65 (85, 86). Airway management and management of inhalation injuries can be challenging in many LMICs where access to intensive care resources like mechanical ventilation and bronchoscopy is limited. Carbon monoxide and cyanide poisoning can be managed with administration of supplemental oxygen (84). Burn wound care is particularly challenging in low resource areas where adequate access to clean water is often problematic. Daily cleaning with Dakin's solution helps to ensure sterility of the water and is bactericidal against most bacteria in the wound (84). The burn wounds are then dressed with antibiotic incorporated dressings (84). The antimicrobial agents used include silver sulfadiazine, mafenide acetate, silver nitrate, and even medical grade honey (84). The need for adequate analgesia cannot be overstated. Opioid analgesia for pain control and ketamine to facilitate wound care are key adjuncts (84). Fevers are common in burn patients but there is no evidence that supports the routine use of prophylactic parenteral antibiotics in the absence of clear evidence of infection (87). In addition, burn centers are more likely to employ contracture avoidance techniques like splinting and physiotherapy to prevent further morbidity.

However, the importance of early recognition, resuscitation initiation, maintenance of euthermia, and basic wound dressings can and should be initiated at the first point of contact. Early referral to tertiary burn centers in LMICs whenever possible is key. Burn prevention and education continue to lead the way in reducing the morbidity and mortality. The majority of childhood burns occur in the home, and therefore public health interventions that target changing the type of fuel used for cooking/lighting, location of these fires, and storage of the fuel have preliminarily resulted in a reduction of burns (88).

### Disasters: pediatric implications

While disasters are often unpredictable, they are neither a fixed singular event nor are they all sudden onset events (89–91). What differentiates them is the scale and magnitude of the impact on families and communities (90, 92). Disasters are increasing in frequency intensity including climate change and environmental degradation related disasters (93). How well families, communities, and government systems prepare for and respond to disasters, determines response and recovery (94). Allocation of technical, financial, and personnel resources during a disaster life cycle (mitigation, preparedness, response, and recovery) is critically important to minimizing mortality and morbidity in extreme events. LMICs are often resource-strapped from poor governance or narrow resource options, and this results in minimal support for disaster cycle provision. Socially vulnerable populations are the most affected by the failure to support disaster cycle planning and implementation (95–97). Children below age 18 and in particular those with disabilities are an especially vulnerable population during and after disasters (95–100).

While the Convention on the Rights of the Child supported by majority countries commit to the right of protecting children from unsafe environments, injury, and violence, the reality in LMIC's and disasters is quite different than envisaged (98, 101). During and after disasters children's needs and protections are more an afterthought, despite the reality that children experience disasters uniquely (102). Children are more susceptible to disaster trauma because of their dependence on adults for information, decision-making, transportation, protection from abuse, and provision of mental support. The assumption that adults promptly inform and make decisions for children in disasters overlooks the reality that children spend substantial amounts of time alone or with their peers or are homeless children living without adults (103). The number of children affected by disasters is projected to triple in coming decades due to factors such as climate change, which UNICEF refers to as a “threat multiplier that exacerbates inequality of children” (93).

In disaster situations, adults, family members, and caregivers are the first responders to children. This is especially true in LMIC's where lag time between disaster impact and response by official responders is substantial or non-existent. Additionally, in complex emergencies population displacement, collapse of health systems and inaccessibility may result in an even larger response lag. Consequently, child-focused training and exercise drills are imperative for mitigating disaster-related injuries or aggravating existing injuries.

### Disaster preparation

Integrating pediatric disaster planning into regular child injury prevention programs is also beneficial to families and communities. Disaster planning learned at school, and other institutions by children benefit families and communities, e.g., who do not speak the dominant disaster planning language (99, 103).

In complex emergencies, mortality rates for unaccompanied minors at refugee camps or shelters increase. Immediate triage and trauma care during intake remain critically important for saving impacted children's lives. Post evacuation to shelters, reunification protocols to protect children from the high risk of abuse, victimization, and trafficking minimizes additional trauma (104). Expeditious reunification (102) to legal guardians is critically important for the commencement of the child's recovery process. Place attachment is a critical component in the recovery processes of children. If evacuation is necessary, or stays in locations other than their places of attachment, creating new place ties bolsters recovery and resilience (95, 98, 105).

### War and its effects on children

**Figure 5:**
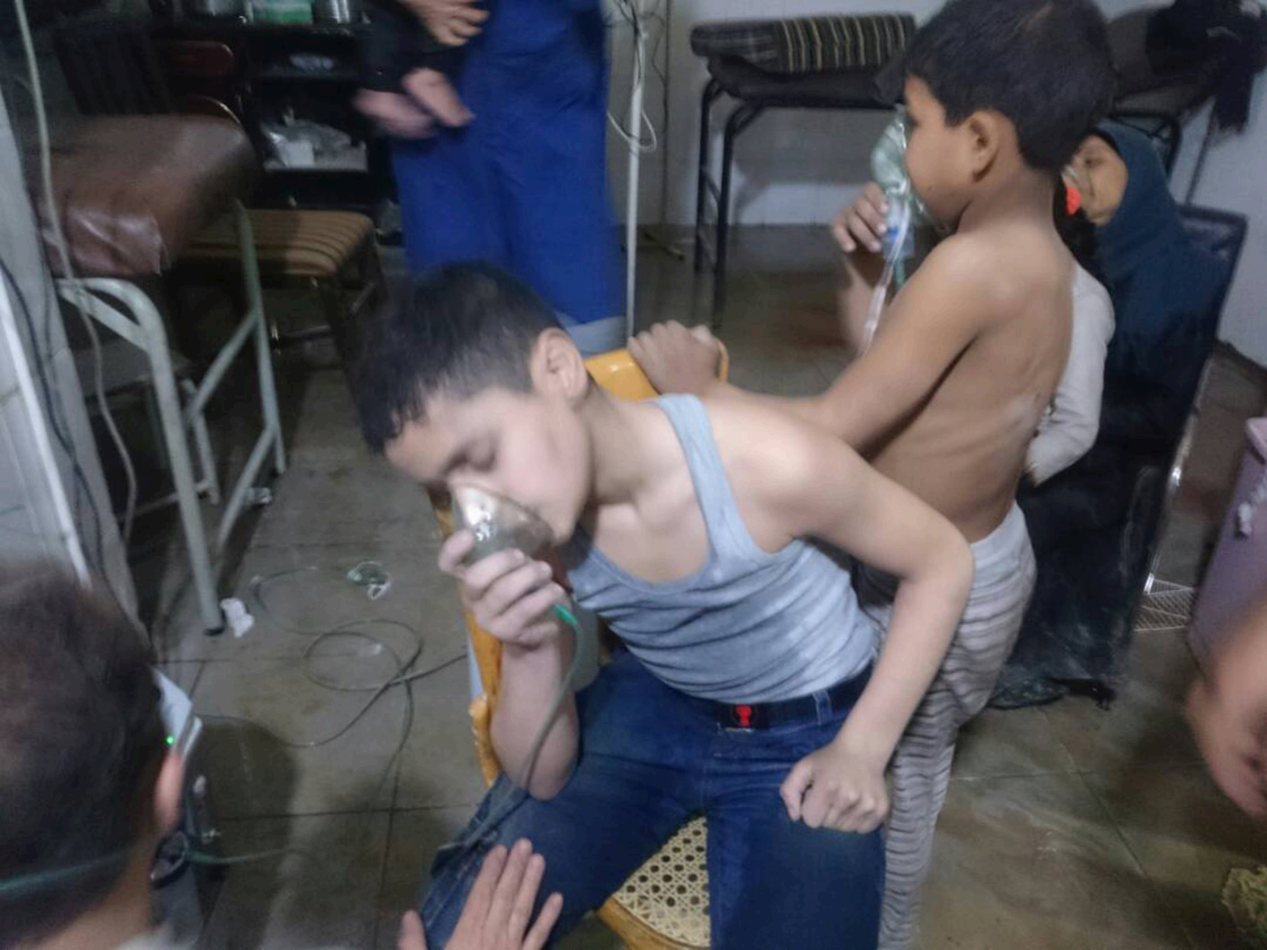
Children being treated after suspected chemical weapon attack in Syria. Image courtesy of Muhammad Ghbeis, Boston Children's Hospital.

Armed conflicts have been and remain part of the very fabric of human history. All around the world wars between nations, civil war, acts of terrorism and other forms of armed conflict persist. The United Nations Children's Fund (UNICEF) estimates that 10% of the world's children (almost 250 million) live in regions affected by war and other armed conflicts (106). The majority of these conflicts are in LMICs (106). Of major concern is the fact that modern warfare is increasingly having a significant impact on the lives of children worldwide (106, 107). The violence is often indiscriminate. There is frequently no defined battlefield, and in most modern conflicts, civilians are directly targeted leading to a marked increase in pediatric injuries and deaths (107). Millions of children have been disabled or killed by this indiscriminate use of force (107). Notably, from 2005 to 2015 the average number of 0–19 disability-adjusted life years (DALYs) due to war and legal intervention increased by 582% (3). The conflicts in Iraq and Afghanistan and in particular the ongoing conflict in Syria, paint a stark portrait of the effects of war on children (Figure 5) (7, 108). Also quite troubling in a number of conflicts, has been the use of children as child soldiers and suicide bombers (106, 107). In addition to physical injuries, psychological wounds including post-traumatic stress disorder and other emotional and behavioral problems result from children's exposure to war (108). Children are also impacted by the disruption to the access to care caused by the conflict and the disruption that additionally results when they become refugees (106). According to the United Nations High Commission for Refugees (UNHCR), by 2015 there were over 65 million people displaced from their homes as they attempted to escape armed conflicts in their countries. About 50% of these are children under the age of 18 (106). It is imperative that children and other civilian non-combatants are protected and that governments and their international partner organizations make every effort to prevent armed conflicts and help end existing conflicts quickly. It is also important that the rights of children are recognized and that those who target children or use them deliberately in armed conflict are brought to justice.

### Child abuse

Child maltreatment occurs in all human communities and across all financial, ethnic, and religious boundaries (109). A recent systematic review of the literature by Hillis et al. found that the actual prevalence of violence against children is higher in LMICs than HICs (59% vs. 44%) (110). This same article reports a minimum of 64, 50, and 34% of children (2–17 yo) in Asia, Africa, and Latin America experienced past-year violence respectively (110). Globally, in 2014 at least 1 billion children were exposed to violence (110). The WHO Global Status report stated that in selected African countries one in three girls are victims of childhood sexual abuse and up to 76% of childhood physical abuse in both boys and girls (111).

A group of children at particularly high risk of abuse in LMICs, are those with disabilities. These children are often socially excluded and prevented from attending schools and are often unable to communicate the violence against them (112).

According to the WHO report, the knowledge of the true extent of the problem is hindered by gaps in knowledge with much of the data coming from high and middle-income countries (111). According to the WHO, a meta-analysis of global data finds self-reported child sexual abuse 30 times higher and physical abuse 75 times higher than official reports would suggest (109).

### Recognizing child abuse

**Figure 6:**
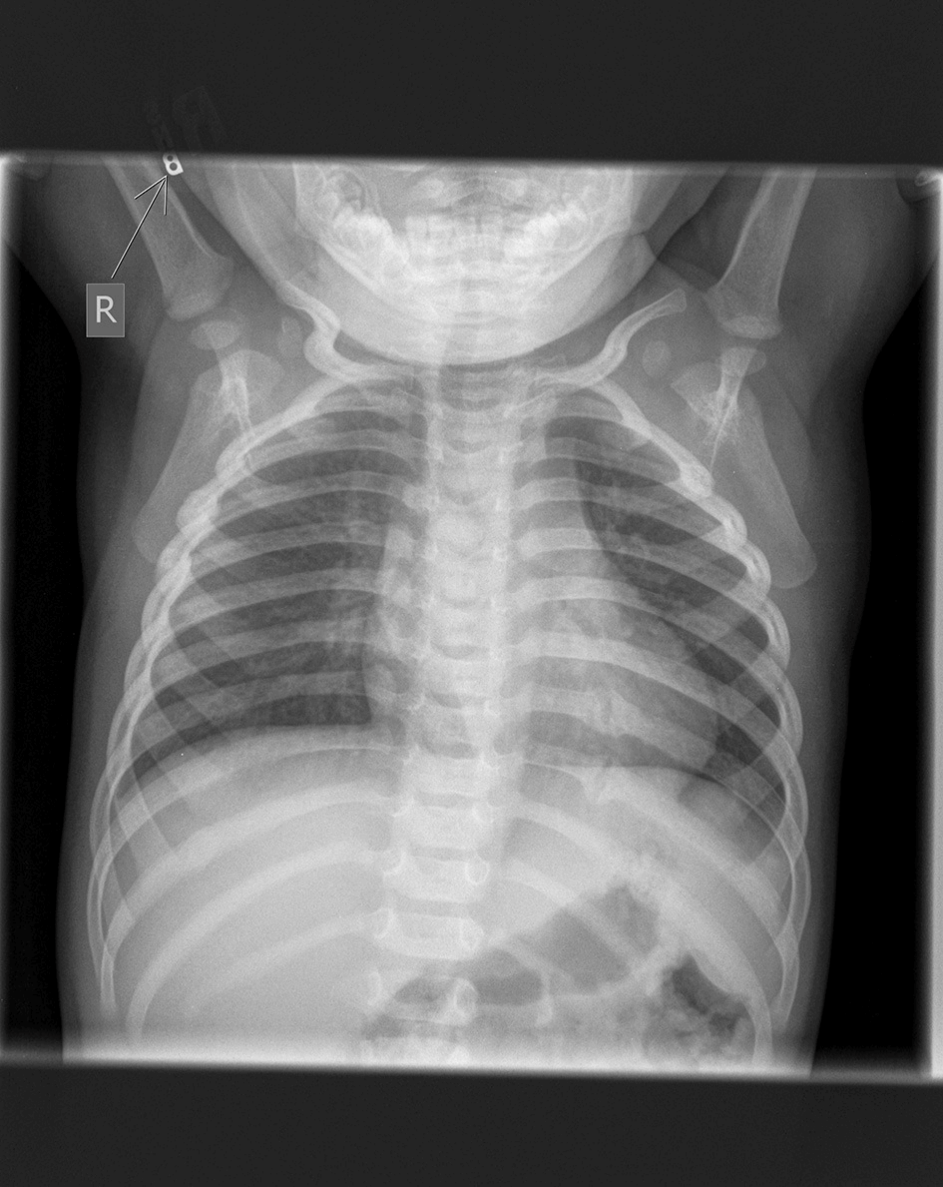
Chest x-ray showing multiple rib fractures from child abuse. Fractures of ribs 3, 4, and 10 on the right and ribs 8, 9, and 10 on the left. Image courtesy of Nancy Harper, MD Masonic Children's Hospital, Minneapolis, MN and Sonja Eddleman, RN, Driscoll Children's Hospital, Corpus Christi, TX.

**Figure 7:**
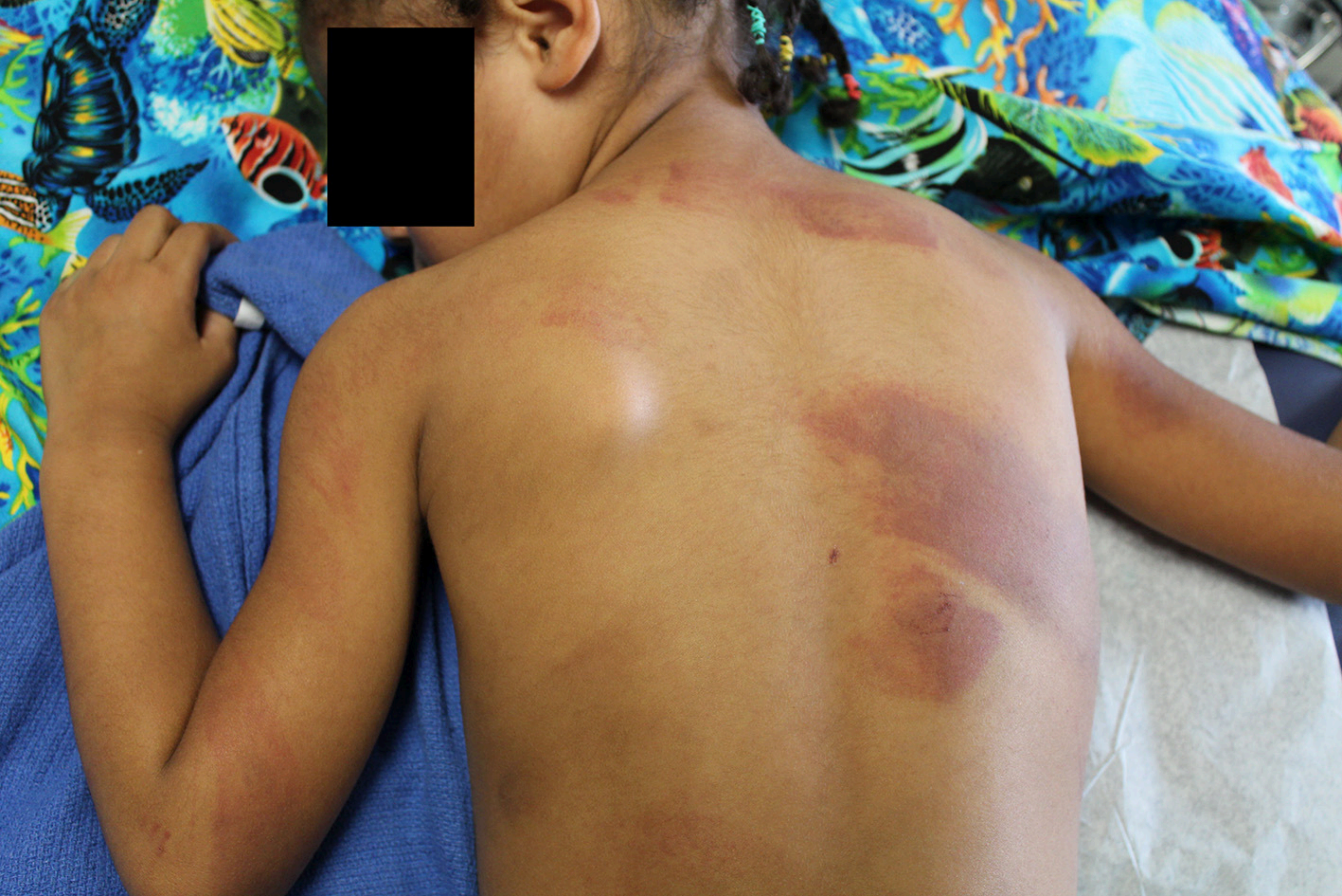
Child with multiple bruises from a belt. Image courtesy of Nancy Harper, MD Masonic Children's Hospital, Minneapolis, MN and Sonja Eddleman, RN, Driscoll Children's Hospital, Corpus Christi, TX.

Although the prevalence and type of abuse may vary by location, the signs and symptoms are similar. We must consider abuse in all children presenting to us with symptoms consistent with abuse such as the triad of retinal hemorrhages, subdural hemorrhage, and encephalopathy or evidence of diffuse axonal injury (113). It is important to note that abusive head trauma can sometimes present with much more subtle findings such as vomiting (113). Additionally, child abuse must be considered in children presenting with features not consistent with the history provided, such as rib fractures from wrestling with another child, or bruises on the ears, under the toes, or in the form of a handprint (111). A helpful mnemonic for assessing which bruises are more concerning is the “TEN 4” rule (Torso, Ear, Neck and 4 (<4 yo or any bruising <4 mo) (113). Worrisome fractures include posterior or lateral rib fractures, “bucket handle” fractures and fractures such as sternal, spine, and scapula (113) unless the child has been in a major motor vehicle or similar accident. (Figure 6) In children, with elevated liver function tests, pancreatic enzymes or otherwise unexplained hematuria abuse with abdominal trauma should be considered (113). Other signs and symptoms that should trigger a careful exam include unusual scars in the form of hand and belt prints, cigarette burns, burns in an unusual distribution, or swelling that is unexplainable such as swollen painful thigh in a young infant (114). (Figure 7) Each of the seven strategies in the INSPIRE strategies, elucidated below are important in prevention as well as specific treatment for the injuries sustained (109, 113). It is also important to differentiate abuse from traditional treatment and practices such as tattooing, cupping and coining prevalent in many cultures (115).

While viewed by many as abusive, female circumcision continues to occur (116). In cultures where this is considered the normal practice, widespread education and laws to protect girls against this practice are needed (116). Another challenging area of child maltreatment is the use of child labor. UNICEF estimates that as many as 246 million children are engaged in child labor with 70% working in hazardous conditions^1^. Child labor may expose children to physical and traumatic maltreatment whether they are involved in hawking wares on the streets, hired as domestic workers or working in factory production lines.

Sexual abuse is common in all cultures but as noted above can be a common form of abuse in LMICs especially in girls. Numerous studies highlight significant rates of unwanted sexual encounters in children. An article by Summer et al. notes rates from 11.3–21.5% depending on age range and country (117). It is important to remember that lack of physical evidence of sexual abuse does not equate to lack of sexual abuse. These children need appropriate counseling and treatment (114, 117).

Child soldiering is a terrible form of maltreatment of children and youth^2^. Children are exploited to achieve the nefarious ends of the abusers as they expose them to violence and rape. These children suffer from post-traumatic stress disorder (PTSD) made worse by the lack of supportive mental health services in many LMICs (118).

Cultures differ in their views about what is appropriate discipline. In some cultures, women and children may be viewed as the property of the husband and therefore virtually nothing constitutes abuse. Male abusers in these cultures are viewed as unquestionable authority figures. Changing norms and values as suggested by the INSPIRE strategies, is likely the most important measure to decrease this abuse. However, laws and their enforcement will also be imperative (109).

Education surrounding inappropriate corporal punishment is needed, as is guidance on the use of other appropriate methods of discipline both at home and in school. Guidelines about age-appropriate discipline are also needed (119).

### Prevention

Given the dearth of available resources, the role of injury prevention in LMICs is vitality important. There are significant human and financial implications for reducing the number of people injured and killed around the world. If injury prevention efforts and improvements in trauma care in LMICs led to injury mortality rates similar to those in HICs, 2 million fewer children would die worldwide (120). The WHO in its *World Report on Child Injury Prevention* (6) set recommendations for prevention of injuries around the world:

(a) Integrating childhood injury into an all-inclusive approach to child health and development
(b) Developing and implementing child-injury prevention policies and plans of action
(c) Implementing specific actions to prevent and control child injuries
(d) Strengthening of health systems to address childhood injuries
(e) Enhancement of the quality and quantity of data for child-injury prevention
(f) Defining research priorities
(g) Increasing awareness of and target investments toward child-injury prevention

The recommendations are a foundation for injury prevention efforts. Injury prevention in children is best achieved by blending education, legislation, law enforcement, environmental modifications and the use of safer products and safety devices (117). Governments in LMICs are increasingly aware of the importance of injury prevention. In Vietnam, where motorbikes are the principal mode of transportation, legislation mandating helmet use by all riders and passengers on motorcycles has the potential to decrease morbidity and mortality in motorcycle-involved RTIs (121). There have also been significant impacts on reduction of injuries from RTIs made by the Road Safety in 10 Countries project (2). The SwimSafe Project which is being implemented in Thailand, Bangladesh, and Vietnam, has taught over 525,000 children how to swim (122). In Bangladesh, one of the largest drowning prevention projects undertaken in an LMIC, the Saving of Lives from Drowning (SoLiD) Project, aims to reduce childhood drowning (123). In addition, given the increasing numbers of children who are injured and killed in armed conflicts, it is imperative that governments and international organizations such as the United Nations, make every effort to end these armed conflicts and minimize their impact on children.

## Conclusions

Trauma is a leading cause of morbidity and mortality globally. Children in LMICs bear the greatest burden of unintentional and intentional injury. LMICs often lack adequate resources for managing trauma. Following trauma care protocols and adapting treatment based on local resources is important. Emphasizing injury prevention, regionalizing care and developing centers of excellence through multispecialty collaboration within each country is vital to improving outcomes and lowering trauma-related morbidity and mortality globally. War is increasingly having a devastating effect on children. A commitment by governments in LMICs in collaboration with international health organizations as well as partners in HICs to provide adequate healthcare services to their populations will be a safeguard against the devastation of infectious diseases and will also lead to improved outcomes for injured children.

## Author contributions

AK: Substantial contributions to the conception or design of the work and the acquisition of background articles and topics to be covered; drafted work and revised it critically for important intellectual content; provided approval for publication; agreed to be accountable for all aspects of the work in ensuring that questions related to accuracy or integrity of any part of work are appropriately investigated and resolved. SD, NM, and AA: Drafted work and added content critically for important intellectual content; provided approval for publication of content. SG, MM and DG: Drafted and revised the work and added content critically for important intellectual content; provided approval for publication of the content. TS: Drafted work and revised it critically for important intellectual content; provided approval for publication; agreed to be accountable for all aspects of the work in ensuring that questions related to accuracy or integrity of any part of work are appropriately investigated and resolved.

### Conflict of interest statement

The authors declare that the research was conducted in the absence of any commercial or financial relationships that could be construed as a potential conflict of interest.

## Footnotes

^1^https://www.unicef.org/protection/files/child_labour.pf

^2^https://www.unicef.org/sowc96/2csoldrs.htm
